# Response to: Arblaster G, Buckley D, Barnes S, Davis H Strabismus Surgery for Psychosocial Reasons – A Literature Review. British and Irish Orthoptic Journal 2024;20:107–132

**DOI:** 10.22599/bioj.448

**Published:** 2025-01-24

**Authors:** Garth Johnston

**Affiliations:** 1 Head Optical Services, King’s College Hospital, SE5 9RS Denmark Hill, London, UK

**Keywords:** Squint surgery, reconstruction strabismus surgery, strabismus surgery, psychosocial outcomes of strabismus surgery

## Abstract

Language is important. Words we use can be much more descriptive. We propose using the prefix reconstruction when describing strabismus surgery. This word emphaises that extra ocualr muscle surgery is much more that cosmetic, it is always functional even when restoring facial aesthetics that improve patients confidence and perception fo themselves to others.

Dear Editor,

Re: Arblaster, G., Buckley, D., Barnes, S. and Davis, H. (2024) ‘Strabismus surgery for psychosocial reasons: A literature review’, *British and Irish Orthoptic Journal*, 20, pp. 107–132, Available at: https://doi.org/10.22599/bioj.352.

The article by Arblaster *et al*. (2024) is a timely reminder on the importance of providing evidence for what we do.

The NHS has survived years of austerity, come through COVID, and we continue to learn from scandals that have rocked the foundations of what most of us would define as high-quality care. At the time of writing, we have a relatively new government who are now beginning to put into place the next stage of their NHS plan.

So, it is right that those who control funding ask some fundamental questions. They need to balance the competing demands of cost against health improvement. This is not a subject that has escaped orthoptics. Many of us will remember the impact of a critical report on school vision screening (Snowdon and Stewart-Brown, 1997), I well remember debates on whether squint surgery was just cosmetic and as such should be provided by the NHS. Yet, orthoptists meet patients every day who feel happier in their lives, now they have straight eyes. We know squint surgery is not just cosmetics.

We can balance the scales, a bit back in our favor. I would ask, as recommended by others (Marsh, 2015), that all orthoptists use the term ‘reconstructive strabismus surgery’ in their clinical reports, letters, and published articles. The term ‘reconstructive’ is regularly used by other specialties, where surgery is conducted not for functional reasons. This is a word missing from this article.

Alternative terms are available (e.g., restorative, rehabilitative, or functional surgery) may best describe those who look forward to a reduction in their symptoms and improvement in their binocular vision. Perhaps BIOS could consider updating the list of agreed orthoptic terms, to be included within SNOMED and other clinical dictionaries?

Reconstructive may be a simple prefix, but we should remember that words are powerful—they can give greater meaning and a better appreciation of what we say.

## References

[1] Arblaster, G., Buckley, D., Barnes, S. and Davis, H. (2024) ‘Strabismus surgery for psychosocial reasons: A literature review’, British and Irish Orthoptic Journal, 20, pp. 107–132. Available at: 10.22599/bioj.352 38681188 PMC11049605

[2] Marsh, I.B. (2015) ‘We need to pay heed to the psychosocial aspects of strabismus’, Eye, 29, pp. 238–240. Available at: 10.1038/eye.2014.283 25475233 PMC4330292

[3] Snowdon, S.K. and Stewart-Brown, S.L. (1997) ‘Preschool vision screening’, Health Technology Assessment, 1(8), pp. i–iv, 1–83, PMID: 9483159. Available at: 10.3310/hta1080 9483159

